# Is there respectful maternity care in Poland? Women’s views about care during labor and birth

**DOI:** 10.1186/s12884-019-2675-y

**Published:** 2019-12-23

**Authors:** Barbara Baranowska, Antonina Doroszewska, Urszula Kubicka-Kraszyńska, Joanna Pietrusiewicz, Iwona Adamska-Sala, Anna Kajdy, Dorota Sys, Urszula Tataj-Puzyna, Grażyna Bączek, Susan Crowther

**Affiliations:** 1 Department of Midwifery, Centre of Postgraduate Medical Education, Żelazna 90, 01-004 Warsaw, Poland; 2 Department of Medical Communication, Medical University of Warsaw, Żwirki i Wigury 81, 02-091 Warsaw, Poland; 3 The Empowering Children Foundation, Walecznych 59, 03-926 Warsaw, Poland; 4 Childbirth with Dignity Foundation, Nowolipie 13/15, 00-150 Warsaw, Poland; 5 Department of Reproductive Health, Centre of Postgraduate Medical Education, Żelazna 90, 01-004 Warsaw, Poland; 6 Department of Obstetrics and Gynecology Didactics, Medical University of Warsaw, Litewska 14/16, 00-575 Warsaw, Poland; 7 School of Nursing and Midwifery, RGU University, Garthdee Road, Aberdeen, Scotland AB10 7AQ

**Keywords:** Childbirth, Ill-treatment, Experience of mother’s birth, Violence

## Abstract

**Background:**

Abuse against women in labor starts with subtle forms of discrimination that can turn into overt violence. Therefore it is crucial to work towards prevention and elimination of disrespect and ill-treatment in medical facility perinatal care in which staff allows such abuse.

The aim of the study was to analyze the experiences of women related to perinatal care. Special emphasis was put on experiences that had traits indicating disrespectful and offensive care during childbirth in medical facilities providing perinatal care.

**Methods:**

This was a cross-sectional survey. A questionnaire was prepared for respondents who gave birth in medical facilities. Information about the study was posted on the website of a non-governmental foundation dealing with projects aimed at improving perinatal care. The respondents gave online consent for processing the submitted data. 8378 questionnaires were submitted. The study was carried out between February 06 and March 20, 2018. The results were analyzed using the Chi-square independence test. The analysis was carried out at the significance level of 0.05 in Excel, R and SPSS.

**Results:**

During their hospital stay, 81% of women in the study experienced violence or abuse from medical staff on at least one occasion. The most common abuse was having medical procedures without prior consent. Inappropriate comments made by staff related to their own or a woman’s situation were reported in 25% of situations, whilst 20% of women experienced nonchalant treatment. In the study 19.3% of women reported that the staff did not properly care for their intimacy and 1.7% of the respondents said that the worst treatment was related to feeling anonymous in the hospital.

**Conclusions:**

The study shows that during Polish perinatal care women experience disrespectful and abusive care. Most abuse and disrespect involved violation of the right to privacy, the right to information, the right to equal treatment, and the right to freedom from violence. The low awareness of abuses and complaints reported in the study may result from women’s ignorance about relevant laws related to human rights.

## Background

In 2014, the World Health Organization issued a statement on prevention and elimination of disrespectful behavior and ill-treatment of women in medical facilities. This document calls for action and research to ensure women’s freedom from abuse and violence within perinatal care [1]. The current guidelines, published in February 2018, underline the importance of respectful care for mothers and children, recognizing the positive experience of women as a priority [2].

Several terms and expressions are found in the literature to describe negative experiences during labor and birth: disrespect, misconduct, disrespectful or arrogant care, offensive behavior, abuse or neglect [3–5]. In addition, he concept of disrespectful and abusive obstetric care is now being used (DACF - disrespectful/abusive care during childbirth in facilities) [6, 7]. In recent years, inappropriate behavior towards women in childbirth began to be described and classified as violence, abuse and discrimination [8, 9]. Moreover, human rights are believed to be violated - when there is a maternal perinatal death associated with the possibility of avoiding complications [10].

Bowser and Hill described seven categories of inappropriate behaviors - physical abuse, non-consented clinical care, non-confidential care, non-dignified care, discrimination, abandonment, and detention in health facilities [6]. However, Bowser and Hill’s work has been critiqued. Freedmann et al. point to the fact that the 7-categories described by Bowser and Hill approach has certain limitations, due to the fact that it concentrates on describing the types of abuse that take place in maternity facilities, but does not take into account the wider context [11]. Freedmann et al. propose a definition that considers both the individual experience of degrading or violent behavior towards women and the importance of structural determinants. This approach reflects the complexity of violence in maternity care. It enables opening to perspectives of very different, often antagonized, groups of stakeholders - women, service providers, politicians and a joint debate on how to improve the quality of care.

A systematic review in the area of negligence and violations of childbirth led by Bohren et al. allowed a widening to the typology of these abuses [8]. The review presented a detailed typology that was evidence based and comprehensively illustrated how women in perinatal care facilities can be mistreated on multiple levels: interactions between women and healthcare providers as well as system and organizational failures. The literature describes respectful maternity care (RMC), which is opposed to abusive care during childbirth in facilities. A recent qualitative evidence synthesis performed by Shakibazadeh et al. elaborates further and mentions how respectful care is concerned with being free from harm and mistreatment [12].

There are many studies of violence and abuse in obstetrics in parts of Africa (Tanzania, Ghana, Kenya, Nigeria) [13–16], Latin America and the Caribbean [17], Pakistan [18]. Yet, there is a paucity of studies related to violence and abuse within European perinatal care. Data from several studies, that included 25% European women, inferred that the occurrence of certain forms of violence during labor may be associated with traumatic deliveries [19].

The scale and type of abuse and violence varies depending on the region of the world, culture or social position [20]. Abuses against birthing women start with subtle forms of discrimination and can turn into overt violence [21]. Research shows that minors, unmarried women, migrants or women from minority groups, as well as those with low socio-economic status, are the most exposed to degrading and inappropriate treatment, and women with HIV are particularly vulnerable to unequal treatment [22]. These abuses and acts of violence towards women during perinatal care have consequences. For example, abusive perinatal care is associated with the risk of complications in the mother and child such as uterine rapture, perineal laceration, neonatal mortality [7, 23].

In Poland, there are legal acts pertaining to a woman’s rights when receiving perinatal care provided by gynecological and obstetric hospitals, for example, The Patient Rights and Patient Rights Law [24] and the Regulation of the Minister of Health of August 16, 2018 on the standard of perinatal care [25] - in short called Perinatal Care Standards (PCS). These legal documents focus on protection of human rights and are evidence based, but they do not define instruments for monitoring the degree of compliance with these principles by specific institutions. These standards refer to patient’s rights [in this case women’s rights receiving perinatal care] and actions that guarantee care consistent with these rights. However, they do not refer to the issue of violence and abuse or the definition of these phenomena in the context of perinatal care exercised in Polish healthcare institutions. Unfortunately, Poland neither employs scientific research nor social activities to lessen and mitigate perinatal abuse and violence within its institutions and that this issue be addressed directly as soon as possible. Using the definitions described in the cited literature this study sought to uncover what is the disrespectful and abusive practices in Polish perinatal care through the experiences of women.

### Aim

The aim of the study was to analyze perinatal care related experiences of women, especially focusing on those that have characteristics that indicate disrespectful/abusive care during childbirth in health facilities.

## Method

### Study design

This was a cross-sectional survey. A questionnaire was prepared for respondents that gave birth in medical facilities. The research was a part of the Childbirth with Dignity Foundation project ‘Monitoring on perinatal care’ (by a grant from the Batory Foundation as part of a program Democracy in action, 2016–2018). Information about the research project was presented on the website of a non-government organization focused on improvement of perinatal care. Information about the survey was disseminated in social and traditional media.

### Setting

The study was carried out between February 06 and March 20, 2018. Information about the study was posted on the website of a non-governmental foundation dealing with projects aimed at improving perinatal care. The information about the study included a link to the questionnaire. The respondents gave online consent to continue with data collection.

If a woman was interested in completing the survey, she was asked to enter her e-mail address and consent given to participate in the study. She then received newsletter type e-mail messages including an individual activation link that redirected women to the page with the questionnaire.

### Participants and measurement tool

#### Variables

The research sample consisted of women who declared that they had given birth in 2017 or 2018. Stillbirth was an exclusion criterion for the study.

Prior to the final survey instrument being sent a pilot was carried out. The pilot invitation was announced at the Foundation’s funpage. There were no selection criteria at this stage. A link was sent to women to check the readability and understanding of the questions. After editing any difficult to understand questions the instrument was re-evaluated by women and then validated by a panel of experts, obtaining the result of S-CVI = 0.89 for 30-item scale. The questionnaire comprised of questions related to care before, during and after delivery. Some of the questions were related to the specific mode of delivery (i.e. a whole block of questions for women that delivered by cesarean section).

Differences in the numbers presented in the tables, result from the fact that only a part of the respondents answered a given question depending on the answers given. Some of the questions were not mandatory, the respondents were allowed not to answer them and go to the next part of the survey. This is also one of the reasons for the different sizes presented in the tables.

Women’s experiences related to abuses were described in more depth through use of open ended questions. The respondents were asked for a detailed description of the situation in which they believed abuse took place during their hospital stay. In the case of open questions, which were answered by more than 1000 respondents, the answers were randomly drawn, categorized, coded and analyzed. The coding was done manually by the researcher. All data were coded by the same person to avoid inconsistency in coding (Questionnaire in the Apendix).

#### Participants

16,500 women volunteered to complete the questionnaire and received an e-mail confirming the survey, not all completed the questionnaire. 10,000 questionnaires were collected and 8378 women completed the questionnaires. The content of the completed 8378 questionnaires were subsequently entered into the final database for analysis.

#### Descriptive data

The characteristics of the study group are given in Table 1. The majority were women aged 26–30 years and 31–35 years. Over 75% of respondents had higher education. This is more than in the Polish population where 30.9% women have higher education [26]. In the sample, over 60% of women gave vaginal birth [*N* = 5345], 35.8% had cesarean section [*N* = 3033]. The percentage of cesarean sections in the sample is lower to the percentage of operative deliveries in the population (43%) [27]. Almost 90% of the respondents, who gave natural birth, gave accompanied birth [*N* = 4782]. Half of the respondents live in large cities and aglomerations (more than 100,000 inhabitants), and 17,5% live in villages.

**Table 1:** Characteristics of the studied group (*N* = 8378)

Age	N	%
Below 25 years	917	11.0
Age 26–30 years	3660	43.7
Age 31–35 years	3002	35.8
Above 36 years	799	9.5
Education		
High School or less	1770	21.1
Higher	6608	78.9
Mode of delivery		
Vaginal delivery	5138	61.3
Instrumental vaginal delivery	207	2.5
Cesarean section	3033	36.2
Place of residence		
City above 500,000 inhabitants	2547	30.5
City above 100,000–500,000 inhabitants	1758	20.9
City between 50,000–100,000 inhabitants	1106	13.2
City below 50,000 inhabitants	1501	17.9
Village	1466	17.5

#### Bias

The source of error could be the inability to fully identify the subjects. To reduce this impact, women had to confirm their participation in the study by e-mail before receiving a questionnaire link. This method of data collection was chosen to provide anonymity to the respondents. The important goal of the study was to get to know the personal and sometimes intimate experiences of the subjects. Whilst confidential information was used in the data analysis it was vital to protect the anonymity of participants.

#### Statistical methods

The quantitative data was analysed by a company specializing in social and marketing analysis. In the analysis of results, data were weighed according to the Central Statistical Office data [26]. Marginal weighing was applied for the province and mother’s place of residence (city or village). The results were analyzed using the chi-square independence test and Z-test. The analysis was performed at the significance level of 0.05 in Excel, R and SPSS. The calculations did not consider data gaps.

## Results

Analyzing the results according to the typology proposed by Bohren et al. 81% of patients on at least one occasion experienced violence or abuse by staff during hospital stay [8]. For example, 55% of women reported experiencing at least one medical procedure being carried out on them during hospitalization without their informed consent.

Respondents were asked about staff behavior during their hospital stay (specifically during labor and birth in the in the obstetric department). Not all types of abuse listed in Bohren et al’s typology of mistreatment of women during childbirth were observed in our study [8]. Responses regarding abuse were grouped according to the following themes: verbal and physical, performing procedures without informed consent, other abuse during delivery (including discrimination, stigmatization, defying the right to secrecy/confidentiality, lack of access to professional care, inappropriate relationships between staff and women). 25% of the respondents reported that during hospital stay hospital staff made inappropriate personal comments related their situation and 20% experienced nonchalant treatment by medical personnel. An important aspect of assessing quality of perinatal care is the way informed consent is given by the woman. The data in this study highlighted that Polish women were not provided adequate informed consent for medical and obstetric procedures.

Table 2 presents the percentage of respondents who experienced some form of physical and/or verbal abuse. Table 3 presents the percentage of respondents who were not asked to give informed consent before medical procedures.

**Table 2:** Physical and verbal violence among the respondents (*N* = 8378)

	The number of women who declared that they have experienced a particular behavior (n)	The percentage of women who declared that they experienced a particular behavior (%)
Verbal violence		
Inappropriate comments	2048	24.4
Nonchalant treatment	1697	20.3
Not answering questions/ignoring	1433	17.1
Raising your voice, shouting, disrespectful expressions	1307	15.6
Mocking	843	10.1
Insulting	565	6.8
Blackmailing with child’s health / woman’s health	411	4.9
Physical violence		
The staff would force their legs apart when pushing	233	2,8
The staff tied their legs to the delivery bed	66	0.8
The staff poked her	38	0.5

**Table 3:** Abuse doing things without asking women for permission (*N* = 4616)

Procedure/treatment	Number of women who declared that the procedure was performed (n)^a^	Percentage of women who were not asked to give informed consent for a given procedure (%)
Enema	*n* = 1715	4.3
Newborn vaccination	*n* = 8345	12.4
Shaving of pubic hair	*n* = 1370	17.4
Newborn examination	*n* = 8346	27.3
Induction of delivery	*n* = 2969	27.1
Administration of an oxytocin drip	*n* = 4143	29.1
Episiotomy	*n* = 3309	30.5
Vaginal examination	*n* = 5710	32.9
Newborn drug administration	*n* = 4278	36.6
Insertion of intravenous cannula	*n* = 6213	40.8
Feeding a newborn baby with modified milk	*n* = 5709	43.2
Presence of students during delivery	*n* = 1118	46.1
Newborn bath	*n* = 6718	48.1

^a^ the percentages were calculated based on the number of people who declared that the procedure was performed

Level of education was the variable that differentiated responses regarding giving informed consent. Respondents with higher education significantly reported more concerns in regard to informed consent than those with secondary or lower education. Table 4. Shows the relationship between educational level and informed consent.

**Table 4:** Performing activities without asking women for informed consent depending on the education of the respondents (N = 4616)

Procedure/treatment for which informed consent was not given	Level of education of respondents				
	High School or Lower		University		*p*-value
	(n)	(%)	(n)	(%)	*p* < 0.05
Enema	49	7.6	61	3.2	p < 0.05
Shaving pubic hair	86	22.5	153	15.4	p < 0.05
Presence of students during delivery	147	9.3	369	7.0	p < 0.05
Oxytocin drip during delivery	340	35.5	864	27.1	p < 0.05
Newborn bath	866	44.8	2365	36.7	p < 0.05
Newborn vaccination	282	14.6	751	11.7	p < 0.05

There were other abuses and forms of violence reported in the study. Table 5. presents the remaining behaviors that were classified as abuses. Almost 30% of respondents declared that during hospital stay the staff performed medical procedures that were rough lacking sensitivity. Among them, 71.8% stated that vaginal examinations were not performed gently, 27.4% mentioned episiotomy repairs being uncomfortable, 19.9% reported that latching the child to the breast was not done sensitively, 14.5% complained that intravenous cannula placement was painful.

**Table 5:** Other abuses excluding violence and excluding the lack of consent for medical procedures (*N* = 4569)

Category of abuse	Types of staff behavior	N = number of respondents	Sample size	Percentage
No access to professional care	Undelicate treatment (internal examination, episiotomy repair)	2597	8378	31.0
	No access to lactation consultant	1871	5740	32.6
	No support in breastfeeding	2410	8378	28.8
	No support in dealing with depressed mood	1395	8378	16.6
	No access to epidural anesthesia	878	6744	13.0
Care that violates the right to privacy/confidentiality	Some activities were done without respect for intimacy	1617	8378	19.3
Improper relations between staff and women	Providing information in an incomprehensible way	1432	8378	17.1
	Not showing respect	1368	8378	16.3
	Conversation in a rude and uncultured manner	1190	8378	14.2
	Not giving all the information needed	2954	8378	35.3
Discrimination and stigmatization	The feeling of being discriminated or stigmatized	739	8378	8.8

19.3% of respondents reported that their privacy and intimacy was not properly taken care of during their hospital stay. Such situations included interviewing or performing tests in the presence of third parties (67.8%), leaving the door open (61.2%), too many people, including staff, during examinations or interviews (56.6%), too many students present during examinations (18.7%) and conversations in the presence of other women or their family members (12%).

Some respondents had negative experience related to communication. Most reported that they did not get all the information they needed from the staff. Yet paradoxically, only a small number recognized that the staff did not show them respect or talked in a rude manner.

Women in the study felt discriminated against in some way, the most common reason being the way staff spoke to them (45%), specifically if women were under 18 years old or above 40 years old (25%), state of health, e.g. chronic disease (18%), body weight (14%). It is also worth noting that 1.7% of respondents reported that the reason for inappropriate treatment was the feeling of anonymity during their hospitalization was due to not having a prior professional relationship with staff caring for them, having prenatal care from a doctor hired by the hospital, and no private midwifery services or care after delivery.

Statistically significant relationships were found between abuse and age, place of residence, education, manner of delivery, use of parenting classes, and birth with an accompanying known person (Table 6).

**Table 6:** The dependence of variables on occurrence / experience of abuse (excluding violence) in childbirth (*N* = 6777)

		Abuse (any indication from Bohren)	No abuse	Test
Age (years)	< 25	779 (84.95%)	138 (15.05%)	X2(3) = 28.22 *p* < 0.001
	26–30	2995 (81.83%)	665 (18.17%)	
	31–35	2399 (79.91%)	603 (20.09%)	
	> 36	604 (75.59%)	195 (24.41%)	
Mode of delivery	Vaginal delivery	4082 (79.45%)	1056 (20.55%)	X2(2) = 21.81 p < 0.001
	Instrumental vaginal delivery	183 (88.41%)	24 (11.59%)	
	Cesarean section	2512 (82.82%)	521 (17.18%)	
Place of residence	City above 500,000 inhabitants	1978 (77.66%)	569 (22.34%)	X2(4) = 29.11 p < 0.001
	City 100,000–500,000 inhabitants	1452 (82.59%)	306 (17.41%)	
	City between 50,000–100,000 inhabitants	929 (84.00%)	177 (16.00%)	
	City below 50,000 inhabitants	1234 (82.21%)	267 (17.79%)	
	Village	1184 (80.76%)	282 (19.24%)	
Education	Elementary/Middle School	66 (88.00%)	9 (12.00%)	X2(3) = 8.05 P < 0.05
	Trade School	87 (79.09%)	23 (20.91%)	
	High School	1314 (82.90%)	271 (17.10%)	
	Higher School	5310 (80.36%)	1298 (19.64%)	
Parenting school	Yes	3651 (83.47%)	723 (16.53%)	X2(1) = 39.41 p < 0.001
	No	3126 (78.07%)	878 (21.93%)	
Childbirth with an accompanying person	Yes	4915 (81.60%)	1108 (18.40%)	X2(1) = 7.06 *P* < 0.01
	No	1862 (79.07%)	493 (20.93%)	

Despite the extent of abuse and violence experienced by women in this study it is concerning that only 15.5% of the respondents recognized that during hospitalization their rights had been violated, moreover, only 3.0% complained that their rights were violated according to the law.

It cannot be ruled out that study participants had strong experiences related to childbirth (both positive and negative) or those who were convinced that participation in this type of research could make a real difference in the care system. At the same time, it should be noted that the majority described their experience of labor as intermediate (neither definitely negative nor definitely positive).

## Discussion

In the Central and Eastern European (CEE) region, the results of prenatal and neonatal care often reach satisfactory levels [28]. In Poland the perinatal mortality of children is 4.9‰, and perinatal mortality rate of women (pregnancy, labor, puerperium) is 0.23‰ (9 cases) [29]. Research analyzing subjective assessment of perinatal care by laboring women, highlights that 30–60% of women find care to be satisfactory [30, 31]. However, it is believed that obstetrical violence globally remains for the most part invisible, due to being part of a cultural context and societal acceptance that forms of violence against women does not necessarily constitute a serious breach of human rights [32]. This may explain the discrepancy between the subjective assessment of care by women, and the incidence of documented violent behavior [11].

In Poland, many changes in perinatal care occurred in the 1990s, after the beginning of social action for dignity in childbirth. In 1994, the “Childbirth with Dignity” campaign was carried out for the first time. It was met with great enthusiasm from women eager to talk about childbirth experiences. Until today, non-governmental organizations play a significant role in assessing the quality of perinatal care in Poland. They conduct numerous educational activities for medical staff and women and periodically monitor perinatal care. Public administration institutions do not maintain a register of data on the quality of care for woman in labor. Institutions are only required to record data on perinatal mortality, financial status of medical services, and the percentage of cesarean sections.

It is difficult to compare the overall level of violations from this study with other studies due to different research criteria and study instruments Although in Poland there has been reports of situations described by Bowser, such as sexual violence, delivery without attendant or dirty beds on the obstetrics ward [6], our study found that the majority of reported abuse was associated with lack of informed consent for medical and obstetric examinations or treatment. An Hungarian study examined consent for performing an episiotomy and found that 62% of Hungarian were not asked for informed consent prior to receiving an episiotomy [33, 34], in our Polish study this percentage was 30.5%.

It is difficult to compare the overall level of violations from this study with other studies due to different research criteria.

Payment for care processes vary across studies. Research in Serbia, Ukraine and Hungary indicate an informal system of fees for the choice of a doctor during labor, helping to facilitate more dignified care [34–36]. In our study 9.8% respondents declared that they had to pay during the stay in a hospital. Respondents stated that lack of additional services (one on one care service) was the reason for abusive treatment. 32.1% of women therefore paid for the presence of a midwife of their choice during delivery.

Freedman describes violent behavior resulting from systemic conditions. According to the regulations in Poland, every woman should have access to this type of epidural anesthesia. However, our study highlighted that was not occurring for all women, for example, no access to epidural anesthesia on demand was reported. It is not known if the lack of access to anesthesia was a result of organizational problem within the medical facility or because of medical contraindications to epidural that were not adequately explained to the woman. Moreover women in our study reported laboring and delivering in shared hospital rooms lacking respect for their privacy [11].

In our study, 16% of women report that the staff are rude to them. This is reflected in other studies. A qualitative review found that women expect a safe, supporting, respectful and responsive care during childbirth [37]. Fear of staff being distant, insensitive or rude has been the subject of many publications [38, 39]. Incorrect communication with staff has also been observed in research in Serbia [35].

It is concerning that insensitive and disrespectful behaviors are reported widely in the literature. Violence and abuse during childbirth are a breach of certain human rights [40]. From this perspective, most of the abuse in our study is associated primarily with violation of the right to privacy, the right to information, the right to equal treatment and the right to freedom from violence. Low awareness of the experienced abuse and the complaints reported in the study may result from the ignorance of women regarding the relevant laws and regulations. A finding in a recent study on Polish women using Scottish maternity services highlights how a social model of care based on relationships and communication was more evident in the UK than in Poland [41]. Women in the Scottish study perceived maternity care to be more medically dominated and the provision of choice considerably less in the Polish system. Women in the Scottish study were surprised at the approach adopted in the UK and expressed surprise at not being told what to do and anticipated more medical procedures. A reason for these perspectives may be due to the intergenerational views about childbirth in Polish hospitals. The experiences of the mothers and grandmothers of women today would not have questioned the rights of the patient and the quality of perinatal care. Some contemporary women may therefore be convinced that, compared to the stories they heard, the care they received was of a high standard. This intergenerational discourse warrants further research and a national campaign to raise awareness across Polish society about the rights of women to receive care that is delivered with sensitivity, tact and gentleness.

### Strengths and limitations

The strength of the study is the size of the studied population. Another strength is the division of answers based on stages of care during hospitalisation. The inclusion of qualitative assessment of violations reported by women was also an important asset. The online data collection approach was helpful because mothers with young children are usually active on the Internet, and an online survey gave them the opportunity to take part in the study at a convenient time, and to interrupt it even when the child was crying and then continue later. Limitations include the tested sample was very large, but not random, so it is not representative. Also, women who participated in the study self-selected and thus wanted to share their experiences and this may have introduced bias. This was also reflected in the over-representation of people with higher education, perhaps caused by the online data collection method. In addition those women who have no access to computers and internet services were silent in this study. The purpose of this work was to show the women’s view of perinatal healthcare, but examining the perspective of medical staff and including observational data could verify the obtained data.

## Conclusion

This study shows that during perinatal care women in Poland experience disrespectful care in health facilities. In Poland it is very common not to ask for permission for various steps of birth related procedures, therefore it was unsurprising that the majority of abusive behaviors reported by women is the failure of hospital staff to explain procedures and obtain informed consent prior to medical procedures. This may explain why the vast majority of Polish women are satisfied with care and do not notice the legal violation of this behavior. Consequently few women decide to file a complaint against hospital staff. This study also highlights the socioeconomic and educational inequalities within Polish perinatal services by foregrounding how women with higher education are more frequently asked for permission and give informed consent to medical procedures.

## Data Availability

The datasets used and/or analyzed during the current study are available from the corresponding author on reasonable request.

## Abbreviations

CEE: Central and Eastern European
DACF: Disrespectful/abusive care during childbirth in facilities
HIV: Human Immunodeficiency Virus
PCS: Perinatal Care Standards
RMC: Respectful maternity care
